# Side Effects following School Deworming among School-Age Children in Oti Region, Ghana

**DOI:** 10.1155/2024/9924852

**Published:** 2024-08-05

**Authors:** Jean Claude Romaric Pingdwindé Ouédraogo, Adolphina Addoley Addo-Lartey

**Affiliations:** ^1^ Department of Epidemiology and Disease Control University of Ghana School of Public Health, Legon, Accra, Ghana; ^2^ Laboratory for Research and Development in Phytomedicines and Medicines (LR-D/PM) Research Institute of Health Sciences (IRSS/CNRST), Ouagadougou, Burkina Faso

## Abstract

**Background:**

Preventive chemotherapy with anthelminthic drugs is meant to control soil-transmitted helminthiases, but some children may experience adverse reactions.

**Objective:**

This study investigated why some school-age children did not receive the medication as well as the side effects experienced by those who did during the 2019 preventive chemotherapy in Krachi East Municipal, Oti Region, Ghana.

**Methods:**

Using a two-stage stratified sampling, a community-based cross-sectional study was conducted among 352 school-age children and their caregivers living in three urban and five rural communities.

**Results:**

Most children (93.8%) were in primary school, aged 11 to 12 years (28.1%), male (53.1%), and resided in an urban area (83.8%). Due to concerns about side effects (28.1%), absenteeism (25.0%), and sickness (9.4%), 32 (9.09%) children did not receive the anthelminthic medication. Of the 320 children who received and ingested the anthelmintic drug, 50.3% experienced at least one side effect. Common side effects included dizziness (58.4%), feeling weak (27.3%), and stomach issues such as vomiting (17.4%), abdominal pain (11.8%), and nausea (6.2%). In adjusted analyses, children aged 11-12 years had higher odds of side effects (aOR: 2.40, 95% CI: 1.22–4.76) than children aged 7-8 years. Male children were also less likely than female children to experience adverse effects (aOR: 0.43, 95% CI: 0.27–0.68). *Discussion*. Ghana's national goal of 100% therapeutic coverage was unmet. Medication consumption during prophylactic chemotherapy may be hampered due to the high prevalence of side effects among school-age children. It is necessary to educate caregivers on how to handle these negative effects.

## 1. Introduction

Soil-transmitted helminthiases (STHs), a Neglected Tropical Diseases' (NTDs) component, are a major public health issue. Globally, it is believed that approximately 24% of the population is affected [1]. Due to inadequate sanitation and hygiene, the poorest inhabitants in subtropical and tropical regions suffer the most. Three (3) species are highly important regarding public health worldwide. These three most deadly species are roundworms (*Ascaris lumbricoides*), whipworms (*Trichuris trichiura*), and hookworms (*Necator americanus and Ancylostoma duodenale*) [1]. In Ghana, intestinal worms are endemic and one of the ten most common diseases [2–4]. STHs are of public health concern when their prevalence exceeds 1% of the population at risk with a medium to high intensity [5]. Adults in high-risk occupational categories, such as farmers or food vendors, women of reproductive ages, preschool children, and school-age children, are the most vulnerable to soil-transmitted helminthiases [1, 4, 6]. Preschool and school-age children infected with STHs are nutritionally and physically disadvantaged [1, 3].

Periodic school deworming to eradicate infecting worms, case management, hygiene education to avoid reinfection, and improved sanitation to break transmission by lowering soil contamination with infective eggs are all used to reduce STHs [1, 2, 7]. Preventative chemotherapy (PCT) is an effective and cost-effective method for controlling or eliminating intestinal worms. In Ghana, only school-age children are targeted for PCT against soil-transmitted helminthiases. It is carried out yearly using an integrated method that includes Albendazole and Praziquantel and targets both soil-transmitted helminths and schistosomiasis [2, 8]. These integrated actions are part of Ghana's essential NTD control efforts [9]. Ghana achieved around 73.80% coverage of Praziquantel + Albendazole among school-age children in 2017, falling short of the WHO target of 75% [10]. Given global progress toward attaining the WHO's 75% target by 2020, the Ghana Neglected Tropical Diseases Programme set a 100% target by 2020 [2]. To eradicate STH reservoirs in communities, it is critical to maintain high treatment coverage over time [11, 12]. Children's and/or caregivers' knowledge about STHs or the PCT, perceptions of associated benefits or risks, and drug-related concerns, notably the fear of side effects, are among the factors that potentially jeopardize treatment coverage [13, 14].

Although PCT is regarded as safe, some side effects may occur, and previous experiences with side effects may cause more anxiety [14, 15]. In India and sub-Saharan Africa, side effect proportions have ranged from 4.1% [16] to 39.2% [15]. Evidence suggests that persons receiving Albendazole in combination with Praziquantel may experience a greater proportion of adverse effects (49.7%) than those receiving only Albendazole (39.2%) [15]. Given that Albendazole is administered throughout Ghana, high proportions of side effects among children would constitute an enormous challenge for treatment coverage. Although the prevalence of soil-transmitted helminthiases species is widely reported in Ghana [4, 17], studies assessing adverse effects associated with preventive chemotherapy in Ghana are sparse. As a result, this study investigated why some children did not receive the medication, in addition to the side effects encountered by school-age children who took the medication during the 2019 preventive chemotherapy in Krachi East Municipality, Ghana.

## 2. Methods

### 2.1. Study Design

A community-based survey was conducted in July-August 2020. Children aged 7 to 14 who lived in the Krachi East Municipality in November 2019 were recruited. The occurrence of side effects following PCT uptake was the response variable, while sociodemographic characteristics of the children were the exposure variables. STROBE reporting guidelines for observational studies were carefully adhered to in the writing of this article.

### 2.2. Participants and Setting

A total of 352 school-age children and their caregivers living in three urban and five rural communities in the Krachi East Municipality participated in this survey. Krachi East Municipality is one of eight districts in the Oti Region, one of Ghana's 16 administrative regions. Krachi East Municipality encompasses an area of 2,529.4 square kilometers and is bounded to the west by Volta Lake. In 2019, the population was estimated to be 143,098 people dispersed among 301 communities [18]. The annual growth rate was projected to be 2.5% on average. Krachi East Municipality is endemic for soil-transmitted helminthiases and schistosomiasis. To address these conditions, the Municipality conducts integrated preventative chemotherapy using Praziquantel and Albendazole once a year. The Municipality's campaign, which spanned November 4th to 8th, 2019, was evaluated in this study.

### 2.3. Sample Size Determination

To reject the null hypothesis that actual uptake equals 70% (i.e., 5% less than the WHO target of 75%), using a two-sided *α* = 0.05, with a 5% precision, the initial sample size (*n*_0_) of 323 children was obtained. Adding a 10% nonresponse rate (*n*_r_), using this formula: *n*_0_/1 − *n*_r_, the final sample size of 352 children was realized.

## 3. Sampling and Data Collection

The participating communities and children were chosen using a two-stage stratified sampling process. The Ghana Statistical Services categorized the 20 largest settlements as rural or urban, with a cut-off of 5,000 residents [18]. Using simple balloting, three rural (Batorkope, Adumadum, and Kudorkope) and three urban (Dambai, Addo Nkwanta, and Asukawkaw) communities were chosen at random. Two additional communities, Sikape and Jerusalem, were randomly selected from the Island subdistrict, which had the lowest PCT coverage in schools (71.5%) during the 2019 PCT (insert reference). Graduate students were employed as research assistants and received training on the project, data-gathering procedures, and research ethics. The initial study's questionnaires were pretested, and any necessary changes were made before data collection. Questions were mostly asked in a local dialect (Twi) or English at the convenience of the participants. The questionnaires were administered using face-to-face interviews, and all the data were gathered using a smartphone and the KoBoCollect software. Caregivers were contacted at their residences, at a time convenient for them and their eligible children. Children were asked if they received and swallowed the medication they were given. The caregivers responded to the side effects encountered by the child after consuming the medication.

### 3.1. Ethical Considerations

All procedures performed in the study were in accordance with the ethical standards of the institutional and/or national research committee and with the 1964 Helsinki Declaration. The research protocol was approved by the Ghana Health Service Ethical Review Committee (GHS-ERC 045/02/20). Each parent or caregiver consented, and assent was sought for the participation of all children. Parents and caregivers who agreed to participate were required to sign or thumbprint the informed consent document.

### 3.2. Data Analysis

Stata/IC 16 (StataCorp LLC, TX, USA) was used to analyze all data. For categorical variables, frequencies and percentages were obtained. When continuous data were normally distributed, means and standard deviations were estimated; when not normally distributed, quartiles, medians, and interquartile ranges (IQR) were reported. The Shapiro–Wilk test was used to determine the normality of continuous variables at a significance level of 5%. The uptake of PCT was estimated as the proportion of children who received and swallowed the drug compared to the total number of children targeted. Chi-square or Fisher's exact tests were used to assess the relationship between side effects and demographic characteristics of the children. All factors associated with side effects by 10% or more were included in the multivariable logistic regression, and *p* values less than 0.05 were considered as statistically significant.

## 4. Results

### 4.1. Sociodemographic Characteristics

In total, 352 children aged between 7 and 14 years from Krachi East Municipality were included. The sociodemographic characteristics of the children are presented in Table 1. Most children came from the Dambai Community (66.5%) and resided in an urban setting (83.815%). Children's median age was 11 (IQR: 9, 12) years. Most of the children were male (53.1%). Only 2 children (0.57%) were not attending school, while 93.8% were at the primary level.

### 4.2. Estimated Coverage, Reasons for not Receiving the Drug, and Prevalence of Side Effects

Albendazole coverage among school-age children was assessed to be 90.9% (95% confidence interval: 87.4%–93.5%), with 320 children out of 352 receiving and swallowing the medicine during the 2019 preventive chemotherapy in Krachi East Municipality. As shown in Table 2, the main reasons for not receiving the medicine were fear or concerns about adverse effects (9/32) and the child's absence that day (8/32). 161 (50.3%) of the children who received and consumed the medication reported at least one side effect (Table 2). The most common side effects (85.7%) were generic reactions including dizziness and weakness. Other children (36.7%) reported digestive issues such as vomiting, pain in the abdomen, nausea, and diarrhea.

### 4.3. Factors Associated with Side Effects' Occurrence

**Figure 1:**
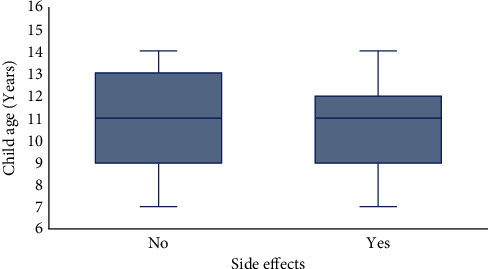
Distribution of children age according to the presence of side effects occurrence following school deworming among school-age children in Krachi East Municipality, Ghana.

Child ages ranged from 7 to 14 years in this population. Among the children without side effects, the median age was 11 (IQR: 9, 13) years. However, the median age was 11 (IQR: 9, 12) years among those with side effects (Figure 1).

From the bivariate analysis, the presence of side effects was significantly associated with the child's age (*p*=0.015) and sex (*p* < 0.001). Among children who reported side effects, most (84.47%) were aged between 9 and 14 years. Other prognostic covariates, such as community, residence, and the child's level of education, had no association with the presence of side effects (Table 3).

The adjusted analyses (Table 4) showed that children who were 11-12 years old had 2.37 times greater odds of reporting a side effect, compared to those 7-8 years old (95% CI: 1.22–4.76, *p*=0.012). Male children also had 57% reduced odds of reporting a side effect compared to the females (95% CI: 0.27–0.68, *p* < 0.001).

## 5. Discussion

The Ghana Neglected Tropical Diseases (NTDs) Programme sought to achieve a treatment coverage of 100% for anthelminthic drugs by 2020. This study assessed the implementation coverage and the occurrence of side effects in Krachi East Municipality following school-based deworming among school-age children in November 2019. The study findings indicate that approximately 1 in 10 children did not receive the drug (Albendazole), mainly due to fear and/or rumors of side effects, child absenteeism, or child sickness. Among the children that swallowed the drug, one-half experienced at least one side effect.

Although the therapeutic coverage of Albendazole was relatively high, above the WHO target of 75% by 2020, Krachi East Municipality failed to reach Ghana's national target of 100% coverage [2]. To effectively control soil-transmitted helminthiases, 100% therapeutic coverage must be achieved and maintained sustainably [19]. However, about 10% of the school-age children did not receive the anthelminthic drug, which could endanger future STH control targets. Children not receiving the anthelminthic drug and swallowing it could make them residual reservoirs of STHs, which can pose dire consequences for all children.

Many reasons were evoked for the school-age children who did not get the drug during the PCT, like the fear or rumors of side effects, child sickness, and child absenteeism. Child absenteeism could be managed by a suitable collaboration between school teachers and children's caregivers. The fear of side effects is the most reported drug-related concern associated with low coverage of anthelminthic treatment against STHs and schistosomiasis [14, 20]. This fear usually emanates from previous experience during preventive chemotherapy against lymphatic filariasis or onchocerciasis using Albendazole and/or other drugs like ivermectin or diethylcarbamazine [14]. The fear of side effects may negatively influence children's adherence and their parents' acceptability of preventive chemotherapy. This would lead to implementation issues, even for the NTD whose drugs have fewer adverse reactions, as preventive chemotherapy is integrated in Ghana [2].

Side effects in the study population were relatively high as 1 in 2 children who swallowed the anthelminthic drug reported adverse reactions. A common conviction is that Albendazole is well tolerated at standard doses, even by young children; and school deworming is well accepted by children, parents, teachers, and health workers [21]. In multicenter studies from India and Haiti, younger children experienced fewer adverse events (14.8%) with Albendazole only [22]. The combined use of Albendazole and Praziquantel during the 2019 preventive chemotherapy could explain the high level of adverse reactions observed in this study. Similar estimates have been reported in Kenya (49.7%) and in Angola (55.9%) among children taking both Albendazole and Praziquantel [15, 23]. Of the school children who took praziquantel alone, up to 49.8% showed side effects [20]. In contrast, few side effects (25.3%) were reported with Albendazole and Praziquantel's coadministration in another study by Njenga et al. among Kenyan school children [24]. This discrepancy may be because participants who were younger than 10 years or older than 14 years were all included in Njenga et al.‘s study [24]. Nonetheless, side effects were more frequent among children aged 10 to 14 years, just as observed in this study. The reported high frequencies of side effects among children aged 7 to 14 years old in Krachi East Municipality could increase the proportion of children not willing to receive the anthelminthic drug in future PCT campaigns, hence reducing the coverage and uptake during such interventions. These implications are not far-fetched as the most common side effects reported were dizziness, weakness, and vomiting, all of which can prevent children from consuming the drug in future interventions. Abdominal pain, dizziness, and vomiting/nausea were also the most frequent side effects reported following the coadministration of Albendazole and Praziquantel in Kenyan school children [15, 24].

The sex of the child was associated with side effects' occurrence in the current study. Essentially, more females experienced side effects even though most of the study participants were males (53.13%). In a study that assessed the risk of adverse swallowing events among school-age children in Kenya, females were also found to experience more side effects than males (60.5% vs 39.5%, respectively; *p*=0.027) [24]. Moreover, females were more prone to experience dizziness than males [24]. In contrast, the risk of adverse swallowing events and choking during deworming was 1.58 (1.24–2.01) times higher among males than female preschool-aged children in Haiti and India [22]. The disparity in findings between continents could be explained by secular differences in the target group (school-aged children).

### 5.1. Strengths and Limitations

This study provides information that can help improve the strategy to control STH in Ghana. However, the study has a few limitations that should be considered. Firstly, this study did not cover all school-age children in the Krachi East Municipality because those aged 5 to 7 years were conveniently excluded. Due to the cross-sectional design of our study, temporal relationships between PCT uptake and side effects cannot be established. Also, only a handful (two) of nonschooling school-age children were sampled in this study, thus limiting the generalizability of the study findings to mostly school-age children who are currently enrolled in schools. Further studies of the coverage of PCT and its side effects should consider more representativeness of such marginal subpopulations as they are also key beneficiaries in national preventative chemotherapy campaigns.

## 6. Conclusion

The preventative chemotherapy for STH control performed well in line with the WHO target but fell short of the national goal of treating 100% of school-age children by 2020. The presence of side effects in one-half of the school-age children could jeopardize PCT intervention's effectiveness. Tailored health education combined with a side effect control system, particularly for children aged 7 to 14 years, will improve preventive chemotherapy outcomes in Krachi East Municipality.

## Figures and Tables

**Table 1:** Sociodemographic characteristics of school-age children in Krachi East Municipality, Ghana.

Sociodemographic characteristics	Frequency *n* (%)
Community	
Dambai	234 (66.5)
Asukawkaw	40 (11.4)
Addo Nkwanta	21 (6.0)
Jerusalem	15 (4.3)
Sikape	15 (4.3)
Kudorkope	10 (2.8)
Adumadum	9 (2.6)
Batorkope	8 (2.3)
Residence	
Urban	295 (83.8)
Rural	57 (16.2)
Child age (years)	
7-8	72 (20.5)
9-10	95 (27.0)
11-12	99 (28.1)
13-14	86 (24.4)
Child sex	
Female	165 (46.9)
Male	187 (53.1)
Child level of education	
None	2 (0.6)
Kindergarten	2 (0.6)
Primary	330 (93.8)
Junior high school	18 (5.0)

**Table 2:** Reasons for not receiving the drug and reported side effects following school deworming among school-age children in Krachi East Municipality, Ghana.

	Frequency *n*	Percentage %
Received the anthelmintic drug		
No	32	9.1
Yes	320	90.9
Reasons for not receiving the anthelmintic drug (*n* = 32)		
Fear of rumors of side effects	9	28.1
Absenteeism of the child	8	25.0
Ill-health	3	9.4
Lack of information	2	6.3
Age or height contraindication	2	6.3
Caregiver did not allow the child	2	6.3
Child is currently not attending school	2	6.3
Other^*∗*^	4	12.5
Swallowed the anthelmintic drug		
No	32	9.1
Yes	320	90.9
Presence of side effects (*n* = 320)		
No	159	49.7
Yes	161	50.3
Reported side effects (*n* = 161)		
Dizziness	94	58.4
Weakness	44	27.3
Vomiting	28	17.4
Abdominal pain	19	11.8
Nausea	10	6.2
Fever	8	5.0
Allergic reaction	6	3.7
Headache	6	3.7
Diarrhea	2	1.2
Other^*∗∗*^	12	7.5

^*∗*^Other reasons: the drug was not administered in that class, drug shortage, did not think it was necessary. ^*∗∗*^Other reported effects: urinating blood, swollen face, itching body, rash, and heartburn.

**Table 3:** Occurrence of side effects following school deworming among school-age children in Krachi East Municipality, Ghana.

	Presence of side effects		*P* value
	Yes = 161 *n* (%)	No = 159 *n* (%)	
Community			0.465
Dambai	106 (65.84)	108 (67.92)	
Asukawkaw	22 (13.66)	17 (10.69)	
Addo Nkwanta	10 (6.21)	11 (6.92)	
Jerusalem	6 (3.73)	4 (2.53)	
Sikape	3 (1.86)	6 (3.77)	
Kudorkope	5 (3.11)	5 (3.14)	
Adumadum	7 (4.35)	2 (1.26)	
Batorkope	2 (1.24)	6 (3.77)	
Residence			0.963
Urban	138 (85.71)	136 (85.53)	
Rural	23 (14.29)	23 (14.47)	
Child age group (years)			0.015
7-8	25 (15.53)	33 (20.75)	
9-10	38 (23.60)	46 (28.93)	
11-12	61 (37.89)	34 (21.39)	
13-14	37 (22.98)	46 (28.93)	
Child sex			<0.001
Female	91 (56.52)	58 (36.48)	
Male	70 (43.48)	101 (63.52)	
Child level of education			0.978
Primary	152 (94.41)	150 (94.34)	
Junior high school	9 (5.59)	9 (5.66)	

**Table 4:** Factors associated with the occurrence of side effects following school deworming among school-age children in Krachi East Municipality, Ghana.

Factors	Simple logistic regression		Multiple logistic regression	
	Crude odds ratio (95% confidence interval)	*P* value	Adjusted odds ratio¥ (95% confidence interval)	*P* value
Age of child (years)				
7-8	1.00		1.00	
9-10	1.09 (0.56–2.14)	0.801	1.10 (0.55–2.19)	0.782
11-12	2.37 (1.21–4.62)	0.011	2.40 (1.22–4.76)	0.012
13-14	1.06 (0.54–2.09)	0.862	1.02 (0.51–2.04)	0.948
Sex of child				
Female	1.00		1.00	
Male	0.44 (0.28–0.69)	<0.001	0.43 (0.27–0.68)	<0.001

¥ Multiple logistic regression: each variable is adjusted for the other variable in the model. E.g., child age is adjusted for sex.

## Data Availability

The dataset is available from the corresponding author upon reasonable request.
